# Group Reminiscence Therapy for Dementia to Improve Well-Being and Reduce Behavioral Symptoms

**DOI:** 10.3390/geriatrics9050109

**Published:** 2024-08-28

**Authors:** Nobuhiko Yanagida, Takumi Yamaguchi, Yuko Matsunari

**Affiliations:** 1 School of Health Sciences, Kagoshima University, Kagoshima 890-8544, Japan; yanagida@health.nop.kagoshima-u.ac.jp (N.Y.); matsuy@health.nop.kagoshima-u.ac.jp (Y.M.); 2 School of Nursing, Tokyo Medical University, Tokyo 160-8402, Japan

**Keywords:** dementia, reminiscence therapy, non-pharmacological intervention, behavioral and psychological symptoms, cognitive function, pre–post comparative study

## Abstract

The global increase in dementia cases highlights the urgent need for effective treatment and care strategies. The aim of this study was to evaluate the effects of group reminiscence therapy on cognitive function, subjective well-being, and behavioral and psychological symptoms of dementia (BPSD) in older adults with moderate to severe dementia. A pre–post comparative design was used, with 49 participants receiving eight group reminiscence therapy sessions over 4 weeks. Baseline, one-week, and one-month postintervention assessments were conducted using the Hasegawa Dementia Scale-Revised (HDS-R), the Neuropsychiatric Inventory-Nursing Home Version (NPI-NH), and the Philadelphia Geriatric Center Morale Scale (PGC Morale Scale). The results showed no significant improvement in HDS-R scores, but significant improvements in PGC Morale Scale (*p* = 0.0417) and NPI-NH scores (*p* = 0.00226), indicating improved well-being and reduced BPSD. These findings suggest that group reminiscence therapy is effective in improving BPSD. Future research should focus on extending the duration of the intervention, including different populations, and combining group reminiscence therapy with other therapeutic approaches to fully determine its long-term benefits and mechanisms. Research on its cost-effectiveness and cultural applicability could further validate and improve the use of group reminiscence therapy in diverse care settings.

## 1. Introduction

The number of people with dementia is increasing rapidly worldwide, making provision of appropriate dementia treatment and care an urgent issue. Currently, 55 million people worldwide have dementia, and approximately 10 million new cases are reported each year [1]. The number of people diagnosed with dementia in Japan is also increasing; there were 4.62 million diagnoses in 2012, 5.17 million in 2015, and 6.02 million in 2020 [2,3,4]. Improving the quality of treatment, providing effective care, and increasing quality of life for people with dementia is essential. Dementia is characterized by core symptoms such as memory impairment, language impairment, and cognitive decline [5], as well as a range of cognitive and behavioral problems classified as behavioral and psychological symptoms of dementia (BPSD) [6]. While dementia encompasses various subtypes with distinct etiologies and cognitive profiles, this study adopted a “global” approach to dementia, focusing on the commonalities in cognitive and behavioral challenges faced by individuals with moderate to severe dementia, regardless of the specific underlying cause. This approach was chosen due to the limited sample size and the focus on evaluating the general effectiveness of group reminiscence therapy in addressing core symptoms and improving quality of life. Dementia not only affects patients, but has physical, psychological, social, and economic effects on their families, caregivers, and society as a whole [7]. The effects of BPSD are a major factor that increases the burden of care, so non-pharmacological therapies for BPSD are considered useful.

Reminiscence therapy was developed by Robert Butler in the 1960s. The therapy is economically attractive because it does not require special equipment or dedicated facilities [8,9]. Trained staff conduct individual or group sessions using specific prompts that evoke the participants’ memories and familiar everyday objects [10]. Previous studies have shown that older adults who participate in reminiscence therapy show considerable reductions in depressive symptoms and substantial improvements in self-esteem, life satisfaction, and psychological well-being [11]. Physiological evaluations following reminiscence therapy have demonstrated changes in brain wave activity, such as increases in alpha and beta waves [12]. Therefore, reminiscence therapy has a range of potential applications as a non-pharmacological therapy, and may improve the daily life of dementia patients, increase their physiological and psychological health, and enhance their sense of purpose and well-being. In particular, reminiscence therapy could help to protect older patients from the substantial side effects associated with pharmacological treatments. However, previous research has focused on the immediate effects of reminiscence therapy interventions, and there is a lack of research on the sustainability and length of the effects. For example, Park et al. (2019) assessed the short-term effects of reminiscence therapy but reported insufficient data on its long-term effects [13].

Therefore, in the present study, we aimed to quantitatively evaluate the effects of group reminiscence therapy on institutionalized older adults with moderate to severe dementia. Specifically, we aimed to evaluate and clarify the effects of group reminiscence therapy on cognitive function, BPSD, and quality of life immediately after the intervention and after a specific period of time. We also aimed to investigate the optimal duration of the intervention and follow-up periods. We hoped to confirm the effectiveness of group reminiscence therapy as a non-pharmacological therapy for patients with dementia and to contribute to improving their quality of life. Our results confirmed the potential effectiveness of reminiscence therapy in improving BPSD in older dementia patients.

## 2. Materials and Methods

### 2.1. Study Design and Setting

We used a pre–post comparative design to evaluate the effects of reminiscence therapy on patients with dementia. Participants were older individuals with moderate to severe dementia who received eight reminiscence therapy sessions over 4 weeks. Evaluations were conducted at baseline (preintervention), 1 week after the last session (follow-up 1), and 1 month after the intervention (follow-up 2).

### 2.2. Contents of Group Reminiscence Therapy

The sessions were conducted by trained staff using specific prompts and familiar objects to evoke participants’ memories. The reminiscence therapy intervention comprised eight elements: birthplace, traditions and customs, school experiences, seasonal changes, books related to childhood, folk tools used in daily life, adolescent life, and old songs such as those from the Ministry of Education songbook or nursery rhymes. Each session was tailored to the personal experiences and preferences of the participants to provide familiar and emotionally engaging topics.

### 2.3. Measurements of Assessment Items and Participants

The assessment indices used were the Hasegawa Dementia Scale-Revised (HDS-R), the Neuropsychiatric Inventory-Nursing Home Version (NPI-NH), and the revised Philadelphia Geriatric Center Morale Scale (PGC Morale Scale). All examinations were conducted by the examiner, an occupational therapist, who visited the facility 1 week and 1 month after the group reminiscence therapy. For the NPI-NH, personal care workers (personal care aides, registered nurses) on the unit served as informants, and the examiner (occupational therapist) interviewed and assessed the informants.

#### 2.3.1. Study Participants

The study was conducted with 49 older dementia patients residing in a nursing home with a specialized dementia unit. The participants were 17 men and 32 women with a mean age of 86.8 years (standard deviation 4.8 years). The inclusion criteria were a diagnosis of dementia and a classification of grade III on the Japanese Ministry of Health, Labour and Welfare’s criteria for the level of independence in daily living of older people with dementia. This classification indicates a condition requiring care owing to symptoms, behavior, or communication difficulties that occasionally interfere with daily life [14]. All participants were aged over 65 years and resided in a nursing home owing to the infeasibility of home care provision. The exclusion criteria included substantial impairment in communication skills, inability to sit for approximately 1 h, and a decision that participation was inappropriate by a patient’s primary care physician.

#### 2.3.2. PGC Morale Scale to Assess Subjective Well-Being

Subjective well-being was assessed using the PGC Morale Scale [15]. This scale was developed by Lawton and measures morale using the subscales of “restlessness”, “attitude toward aging”, and “loneliness/dissatisfaction”. Morale is defined as an attitude and willingness to actively contribute to the achievement of group goals. The lead occupational therapists administered the scale by reading the questions to the participants and recording their responses.

#### 2.3.3. HDS-R for Assessment of Cognitive Function

Cognitive function was assessed using the HDS-R, which assesses memory, attention, calculation, and language skills. The HDS-R consists of 11 items that are scored 0–1 or 0–2; the total score ranges from 0 to 30 with a varying number of possible points, according to the number of correct responses. Scores below 19 indicate dementia, 20–26 indicate mild cognitive impairment, and 27–30 are considered normal. The HDS-R has high consistency and test–retest reliability [16,17] and is widely used in clinical settings. A score of 20 on the HDS-R is used as a cutoff point to differentiate between mild cognitive impairment and more severe dementia. The use of this cutoff ensures consistency with previous studies [16]. The assessments were conducted according to the HDS-R manual.

#### 2.3.4. NPI-NH for Assessed BPSD

BPSD were assessed using the NPI-NH, which assesses 10 items: delirium, hallucinations, agitation, depression, anxiety, disinhibition, irritability, abnormal behavior, nocturnal behavioral disturbances, and appetite/eating abnormalities. Each item is scored on frequency and severity; the total score ranges from 0 to 120. Higher scores indicate greater severity of BPSD [18,19,20]. Occupational therapists involved in the study assessed BPSD by obtaining information from nurses and caregivers who provided daily care to the participants.

### 2.4. Statistical Analysis

To analyze the data, the normality of continuous variables was assessed using the Shapiro–Wilk test and Q–Q plots [21]. Because the continuous variables were not normally distributed, as confirmed by both statistical tests and visual inspection, generalized linear mixed models (GLMMs) were used [22]. The GLMMs included HDS-R, PGC Morale Scale, and NPI-NH scores as dependent variables, number of measurements and age as fixed effects, and participant ID as a random effect. Bolker et al. (2009) and Harrison et al. (2018) [22,23] have demonstrated the appropriateness and robustness of GLMMs even in studies with small sample sizes. Therefore, the use of GLMMs was considered appropriate in this study despite the small sample size All statistical analyses were performed using R software (version 4.3.1), with a significance level of <5%.

## 3. Results

### 3.1. General Results

Table 1 shows the results of the summary statistics. The mean HDS-R score was 9.59 at preintervention baseline, 10.35 at 1 week postintervention, and 9.79 at 1 month postintervention; the mean PGC score was 9.29 at baseline, 9.94 at 1 week postintervention, and 10.41 at 1 month postintervention. The mean NPI-NH score was 22.08 at baseline, 15.8 at 1 week postintervention, and 16.5 at 1 month postintervention.

### 3.2. Change in HDS-R Score for Repeated Measures

Table 2 shows the changes in scores on the HDS-R at baseline, one-week, and one-month evaluation time points. The results showed no significant improvement in scores at the time of the HDS-R assessment (B = 0.2102, *p* = 0.939).

### 3.3. Change in PGC Morale Scale Score for Repeated Measures

**Figure 1:**
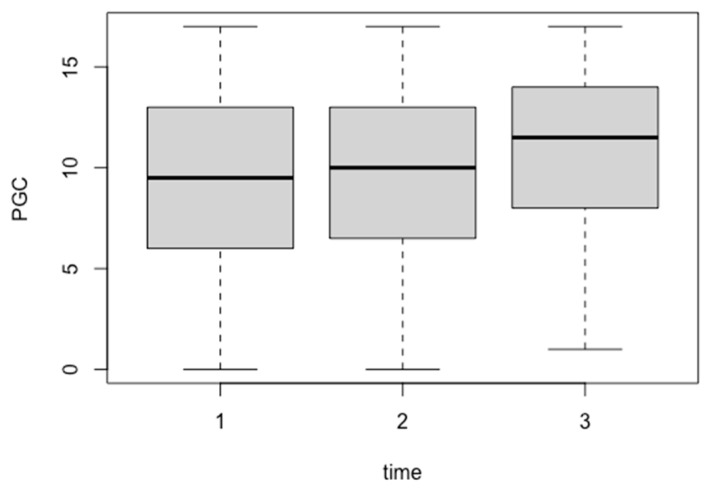
Trends in the revised Philadelphia Geriatric Center Morale Scale (PGC) scores over the course of the study. The *x*-axis represents the number of measurements (baseline, one-week post-intervention, and one-month post-intervention), and the *y*-axis represents the PGC Morale Scale scores.

Table 3 and Figure 1 show the results for the change in scores on the PGC Morale Scale at baseline, one-week, and one-month evaluation time points. The results showed a statistically significant improvement in scores at the time of the evaluation (B = 0.54813, *p* = 0.0417).

### 3.4. Change in NPI-NH Score for Repeated Measures

**Figure 2:**
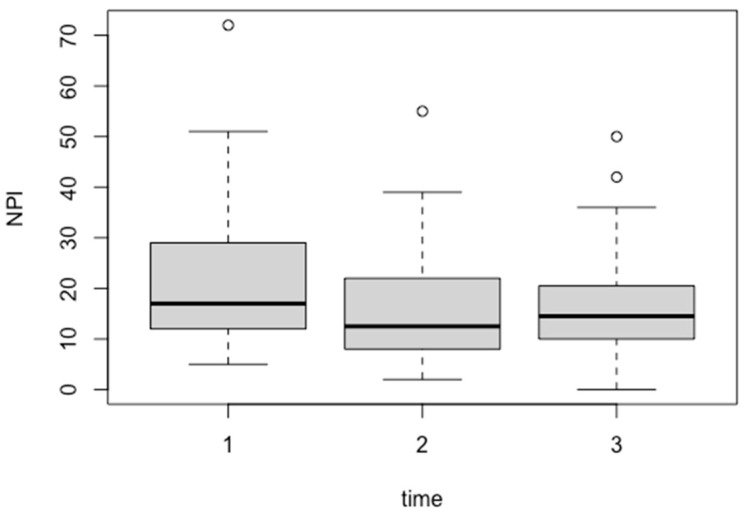
Trends in the Neuropsychiatric Inventory-Nursing Home Version (NPI-NH) scores over the course of the study. The *x*-axis represents the number of measurements (baseline, 1 week post-intervention, and 1 month post-intervention), and the *y*-axis represents the NPI-NH scores.

Table 4 and Figure 2 show the results for the change in scores on the PGC Morale Scale at baseline, one-week, and one-month evaluation time points. The results showed a statistically significant improvement in scores at the time of the NPI-NH evaluation (B = −2.66017, *p* = 0.00226).

## 4. Discussion

The study aim was to evaluate the effects of group reminiscence therapy on older adults with moderate to severe dementia. The results provide valuable insights into the effects of group reminiscence therapy on cognitive function, subjective well-being, and BPSD.

### 4.1. Effect on Cognitive Function

HDS-R scores showed no significant improvement at baseline, 1 week postintervention, or 1 month postintervention (B = 0.2102, *p* = 0.939). This finding is consistent with previous studies showing that short-term cognitive improvements following non-pharmacological interventions for people with dementia are often limited [24]. Cognitive function in dementia patients may require longer and more frequent interventions to produce substantial changes [25]. In addition, the low baseline scores may indicate severe cognitive impairment, which limits the potential for noticeable short-term improvements [26].

Additional research that extends the duration and frequency of the intervention is needed to assess its long-term effects on cognitive function. Combining group reminiscence therapy with cognitive stimulation therapy may also yield better results, as the latter has shown promise in improving cognitive function in patients with dementia [24]. In addition, the inclusion of other cognitive-enhancing activities along with group reminiscence therapy could create a synergistic effect, leading to greater improvements in cognitive domains.

### 4.2. Effect on Subjective Well-Being

Significant improvements in PGC Morale Scale scores were observed at 1 week and 1 month postintervention (B = 0.54813, *p* = 0.0417). These findings are consistent with previous research highlighting the positive effects of reminiscence therapy on the psychological well-being of older adults [27]. Reminiscence therapy facilitates the recall and sharing of positive memories, which increases self-esteem and reduces feelings of loneliness and depression [28]. The social interaction and emotional support provided in group settings further contribute to the overall improvement in morale [29,30]. Reasons why group reminiscence therapy improves psychological well-being include increased self-esteem and reduced feelings of loneliness. 

These improvements in subjective well-being suggest that group reminiscence therapy may be a valuable tool for improving the quality of life of people with dementia. The emotional and social benefits of reminiscence therapy are important given the high prevalence of depression and social isolation among people with dementia [31]. Future studies should explore the integration of group reminiscence therapy into regular nursing home care routines to maintain and potentially increase these benefits. In addition, evaluating the effect of group reminiscence therapy on different dimensions of quality of life, such as social relationships and physical health, could provide a more comprehensive understanding of its benefits.

### 4.3. Effect on Behavioral and Psychological Symptoms

We also identified significant improvements in NPI-NH scores, indicating a reduction in BPSD (B = −2.66017, *p* = 0.00226). This finding supports the efficacy of non-pharmacological interventions, such as reminiscence therapy, in the management of BPSD [32]. The structured and engaging nature of group reminiscence therapy sessions provides cognitive and emotional stimulation that may help to alleviate symptoms of agitation, anxiety, and depression [33].

Reducing BPSD not only improves patients’ quality of life, but also reduces caregiver burden. The present findings are consistent with the theory that reminiscence therapy can serve as a therapeutic outlet for expressing emotions and resolving past conflicts of patients, thereby reducing psychological distress [34]. These findings emphasize the importance of incorporating group reminiscence therapy into care plans for patients with dementia to effectively manage behavioral symptoms. In addition, exploring the specific aspects of group reminiscence therapy that contribute most to the reduction of BPSD could help to refine and optimize this type of therapy and produce better outcomes.

### 4.4. Generalization of Findings

The present findings are encouraging, and suggest that group reminiscence therapy is an effective intervention for improving subjective well-being and reducing BPSD in older adults with moderate to severe dementia. Integrating group reminiscence therapy into dementia care programs in nursing homes and similar settings may improve the overall quality of care. Implementing group reminiscence therapy as part of a holistic approach to dementia care could address not only the cognitive, but also the emotional and behavioral needs of patients [35]. Compared with other non-pharmacological therapies, group reminiscence therapy is particularly effective in generating social and psychological responses. For example, music therapy elicits emotional responses, whereas group reminiscence therapy promotes self-affirmation through the sharing of past experiences [36].

Future research should aim to replicate these findings with larger, more diverse samples and over longer periods of time to confirm the long-term benefits of group reminiscence therapy. In addition, cross-cultural studies would be useful to determine whether the effectiveness of group reminiscence therapy varies across cultural contexts, further broadening its applicability.

### 4.5. Study Limitations and Future Directions

This study had several limitations that must be considered. The sample size was relatively small, which may limit the generalizability of the findings. Future studies should include larger and more diverse populations to ensure broader applicability. In addition, the study duration was relatively short, which limits the ability to assess long-term effects. Longitudinal studies are needed to assess the sustainability of any observed benefits over time [37]. Furthermore, all of this study’s participants had Alzheimer’s disease as their primary disease, and the results do not indicate that group reminiscence therapy is effective for all types of dementia. In addition, exploring the specific impact of group reminiscence therapy on different dementia subtypes, considering their unique cognitive and behavioral profiles, could provide valuable insights into tailoring interventions for optimal outcomes. Therefore, future comparative studies with a control group should be conducted to obtain more robust results. Moreover, our study did not explicitly consider the clinical criteria used in the initial diagnosis of participants. Clinical criteria are of major importance in guiding the etiological diagnosis of dementias, especially Alzheimer’s disease [38]. Future studies should incorporate these clinical criteria more comprehensively, as they may provide valuable insights into the differential effects of group reminiscence therapy across various dementia subtypes and stages of progression.

In addition, the reliance on self-report measures may have introduced bias. Incorporating objective measures, such as neuroimaging or biomarker analysis, may provide more robust data on the effects of group reminiscence therapy [39]. Combining group reminiscence therapy with other therapeutic approaches, such as music therapy or exercise programs, may provide a more comprehensive strategy for managing dementia symptoms [40]. Investigating the cost-effectiveness of group reminiscence therapy and its effect on health care resources could also provide valuable insights for policy makers and health care providers. Additionally, integrating standardized clinical criteria alongside these objective measures would offer a more holistic view of the therapy’s impact, potentially revealing correlations between clinical presentations and therapeutic outcomes.

## 5. Conclusions

In conclusion, our findings are largely consistent with the existing literature, demonstrating that group reminiscence therapy can be an effective non-pharmacological intervention for improving well-being and managing symptoms in older adults with dementia. Although no immediate cognitive benefits were observed, the significant improvements in subjective well-being and reduction in BPSD indicate the therapeutic potential of reminiscence therapy. Additional research with larger samples, longer follow-up periods, and additional objective measures is essential to fully understand the long-term benefits and mechanisms of group reminiscence therapy. Broadening the scope of research to include cost-effectiveness and cultural applicability could further support and improve the use of group reminiscence therapy in diverse care settings.

## Figures and Tables

**Table 1:** Summary statistics for scores on each scale.

Statistics	HDS-R			PGC Morale Scale			NPI-NH		
	Baseline	Follow-up 1	Follow-up 2	Baseline	Follow-up 1	Follow-up 2	Baseline	Follow-up 1	Follow-up 2
n	49	46	47	48	47	44	49	46	44
Mean	9.59	10.35	9.79	9.29	9.94	10.41	22.08	15.8	16.5
SD	5.42	5.86	6.62	4.55	4.33	4.28	14.39	10.59	10.25

Notes. n, number; SD, standard deviation; HDS-R, Hasegawa Dementia Scale-Revised; PGC, Philadelphia Geriatric Center; NPI-NH, Neuropsychiatric Inventory-Nursing Home Version.

**Table 2:** Change in HDS-R scores at baseline, one-week, and one-month evaluations.

	Estimate	Std. Error	*t*-Value	*p*-Value
(Intercept)	32.9108	41.5993	0.791	0.43
Age	−0.2073	0.4744	−0.437	0.663
Evaluation time points	0.2102	2.745	0.077	0.939

Notes. HDS-R, Hasegawa Dementia Scale-Revised.

**Table 3:** Change in PGC Morale Scale scores at baseline, one-week, and one-month evaluations.

	Estimate	Std. Error	*t*-Value	*p*-Value
(Intercept)	4.86173	10.36497	0.469	0.6412
Age	0.04603	0.119	0.387	0.7007
Evaluation time points	0.54813	0.26531	2.066	0.0417

Notes. PGC, Philadelphia Geriatric Center.

**Table 4:** Change in NPI-NH scores at baseline, one-week, and one-month evaluations.

	Estimate	Std. Error	*t*-Value	*p*-Value
(Intercept)	22.48368	27.43798	0.819	0.41675
Age	0.01429	0.31482	0.045	0.96401
Evaluation time points	−2.66017	0.84646	−3.143	0.00226

Notes. NPI-NH, Neuropsychiatric Inventory-Nursing Home Version.

## Data Availability

All data are available from the corresponding author on reasonable request.
